# Spinal Introducer Needle Breakage During Spinal Anesthesia for Cesarean Section: A Case Report

**DOI:** 10.7759/cureus.46972

**Published:** 2023-10-13

**Authors:** Suzana Sobot Novakovic, Dragan Rakanovic, Tanja Milic-Radic

**Affiliations:** 1 Anesthesiology and Critical Care, University Clinical Centre of the Republic of Srpska, Banja Luka, BIH; 2 Obstetrics and Gynaecology, University Clinical Centre of the Republic of Srpska, Banja Luka, BIH

**Keywords:** spinal needle breakage, anesthesia complications, cesarean section, obstetric anesthesia, spinal anesthesia

## Abstract

Spinal and epidural anesthesia are the preferred choices for patients undergoing a cesarean section (CS). The increased use of neuraxial anesthesia in obstetrics may lead to certain complications such as needle breakage. While several cases of broken spinal and epidural needles have been reported, the exact incidence of needle breakage remains uncertain. The use of pencil-point needles with smaller diameters and the increasing BMI among pregnant individuals may have contributed to the increase in the reported incidents of broken needles during obstetric surgery. We present a case of a patient who was found to have a leftover spinal introducer needle in her back after undergoing spinal anesthesia for CS.

## Introduction

Even though spinal anesthesia is generally considered safe for pregnant patients, like all medical procedures, it is associated with certain potential complications. The most frequent of these complications is hypotension, which is usually well managed with medications and fluid administration [1]. Post-dural puncture headache (PDPH) occurs in 1-2% of cases due to the use of pencil-point needles [2]. Other potential complications include infections and nerve damage, but they are very rare and occur approximately in 1/10,000 and 1/100,000 patients respectively [3,4].

Spinal needles are designed and manufactured to be strong and durable. However, factors such as improper use, manufacturing defects, or accidental trauma can potentially lead to needle breakage. The use of proper techniques, high-quality equipment, and adherence to safety guidelines significantly reduce the risk of such incidents [5].

During spinal anesthesia, an introducer needle is commonly employed to provide stability for the subsequent placement of small-gauge and non-cutting tip needles. The blunt tips of non-cutting needles may encounter significant resistance during puncture. To minimize tissue damage and reduce the risk of needle breakage, it is advisable to use an introducer when using non-cutting needles [6]. The introducer helps secure the insertion of the non-cutting needle and can be especially helpful when using fine needles of 25 G or smaller [7]. However, it is crucial to ensure that the introducer is correctly inserted to prevent the pencil-point needle from inadvertently passing through the spinal canal. Although breakage of the introducer needle has been rarely described, it can happen due to a manufacturing error or incorrect use of the introducer needle [8,9].

## Case presentation

A 35-year-old woman was brought to the emergency department (ED) due to back pain following a cesarean section (CS). The CS had been performed under spinal anesthesia seven days prior to her visit to the ED. The patient specifically complained about pain in her back where the spinal anesthesia had been administered. The pain had started right after the surgery when the effects of anesthesia had worn off, and she described it as sharp and intensifying when she moved. Additionally, she mentioned discovering a broken needle part, specifically the needle hub, in her bed during her stay in the hospital after CS, which led her to suspect that a portion of the needle may have been left in her back. In her previous hospital stay, the patient had been examined but not given a proper diagnosis before being discharged. Hence, she had come to the ED seeking further evaluation and a second opinion.

**Figure 1:**
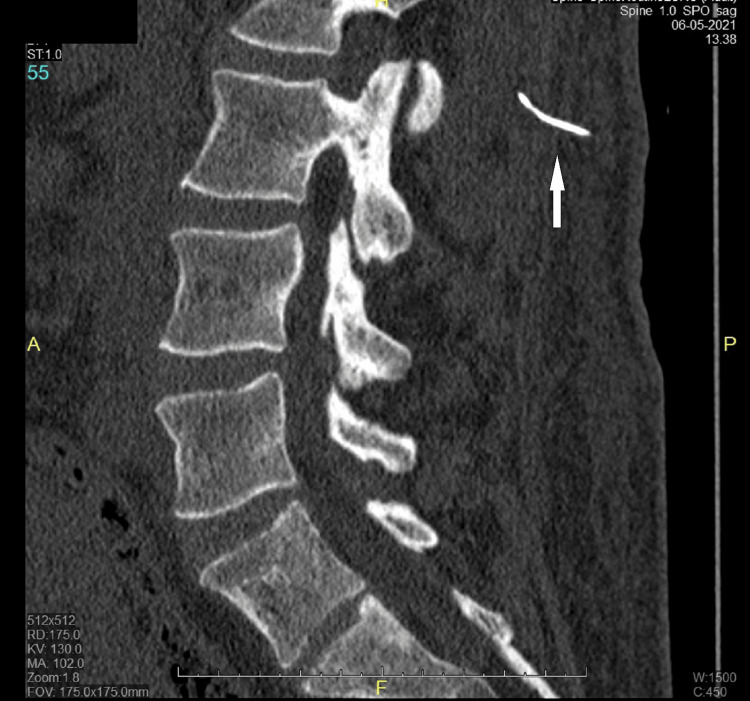
CT scan of the lumbar spine - image 1 The sagittal plane image shows a broken needle fragment (white arrow pointing to the needle fragment) at the L2 level, at a depth of 13 mm from the skin CT: computed tomography

**Figure 2:**
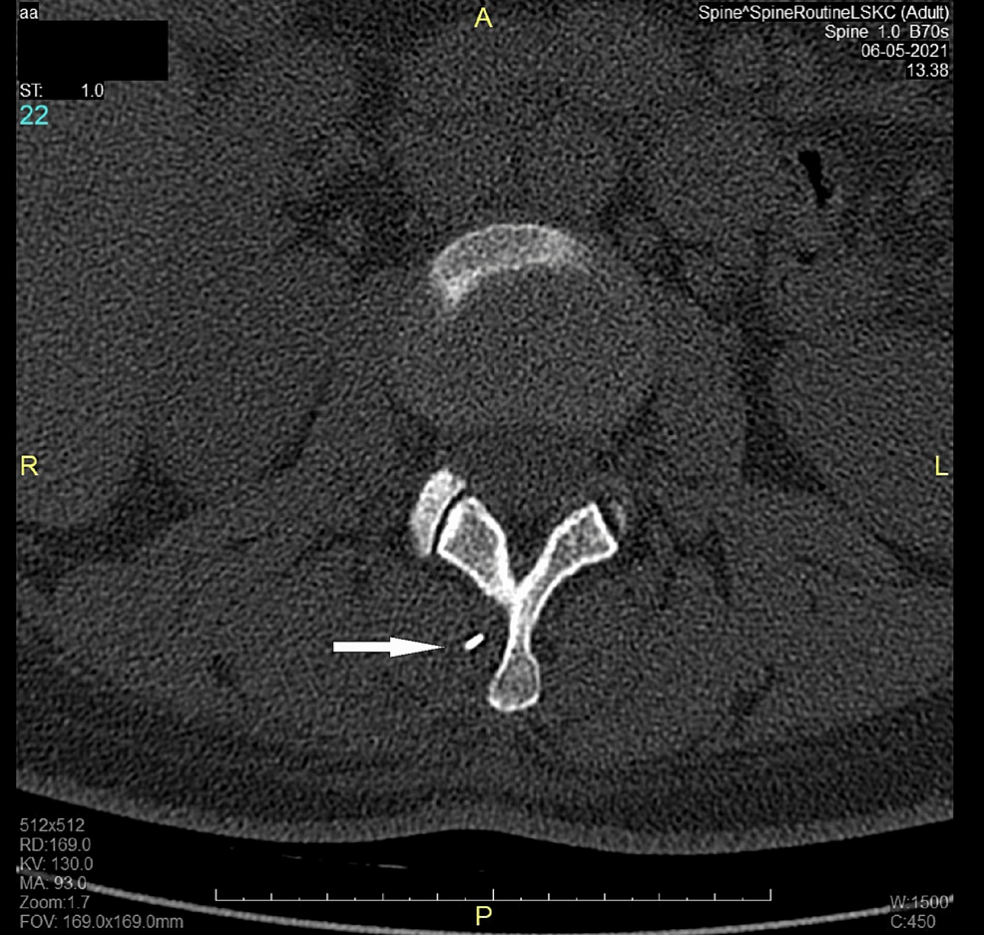
CT scan of the lumbar spine - image 2 The horizontal plane image shows the tip of the needle (white arrow pointing toward the tip of the needle) close to the lateral side of the L2 spinous process, within the right paravertebral space of the erector spinae muscle CT: computed tomography

**Figure 3:**
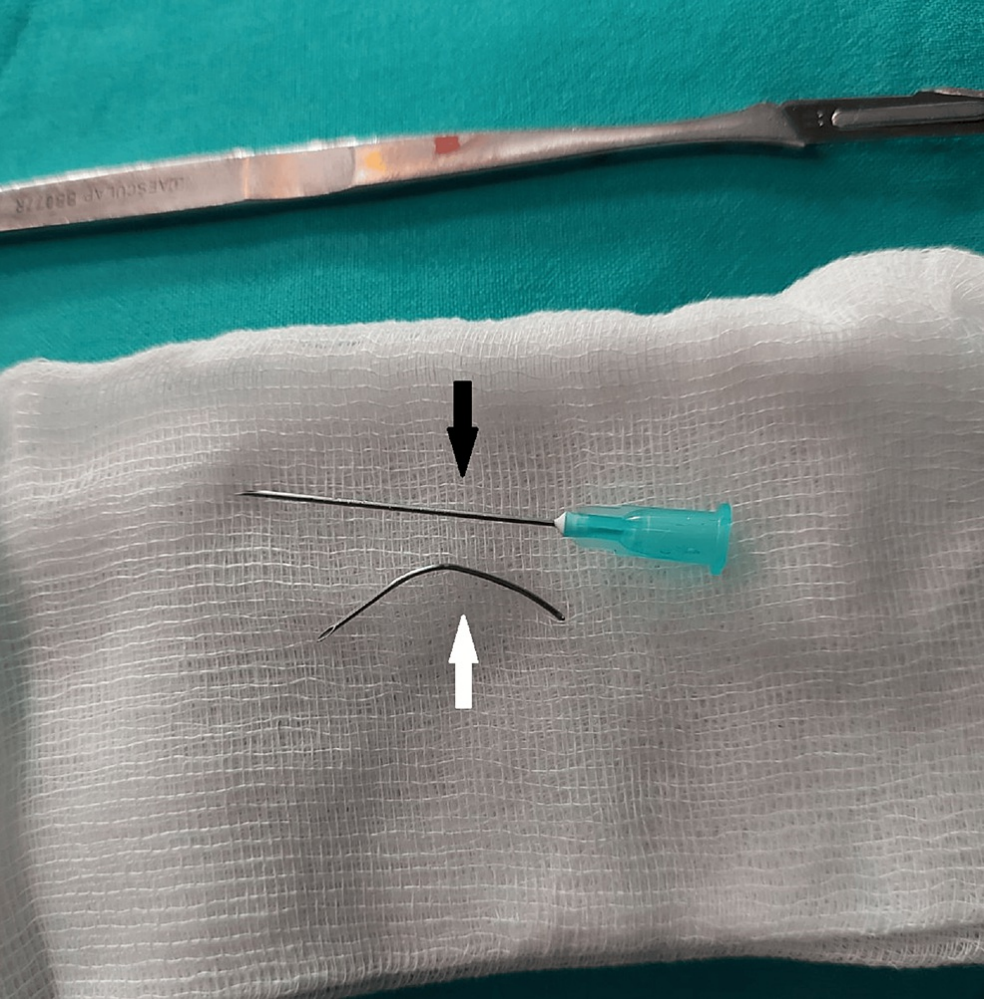
The extracted broken spinal introducer needle The white arrow points toward the deformed broken spinal introducer needle. The black arrow points toward a 21-G, 40-mm injecting needle placed only for comparison

During the examination, a non-inflamed swelling area was observed in the patient's back at the L2 and L3 interspinous level, the area where the spinal anesthesia had been administered. The swelling area was tender to touch, did not fluctuate, and there was a visible mark from the previous needle puncture. Laboratory testing was performed, as well as radiology and neurosurgery consultation. CT scan of her lumbar spine revealed a 2-cm metallic needle fragment around her spine. The needle was found within the erector spinae muscle, on the right side, approximately 13 mm beneath the skin, and about 4 mm from the side of the L2 spinous process (Figures 1, 2). We took the patient straight to the operating room (OR). Removal of the broken needle fragment was performed by a neurosurgeon using local anesthesia. The needle was removed with the help of fluoroscopic control. A 5-cm incision was made in the skin to locate and remove the broken fragment. The broken fragment turned out to be a spinal introducer needle that comes with the pencil-point spinal needle (Pencan®, B Braun, Melsungen, Germany) (Figure 3). After the surgery, the patient experienced relief and was free from pain and neurological symptoms. A follow-up examination three months later showed no symptoms or difficulties. The patient decided to drop the lawsuit that she had filed against the hospital.

## Discussion

The exact incidence of broken spinal needles is not known. According to the literature, the reported incidence ranges from one in 5000 to one in 11000 cases [10,11]. Approximately 50% of the cases of broken spinal needles have been found in obstetrics. Martinello et al. listed 15 relevant case reports in their review article, and of these, seven were related to obstetrics. This could be attributed to the increasing use of neuraxial anesthesia in obstetrics and the higher BMI among pregnant women [11].

There are certain factors that can contribute to needle fractures during medical procedures [5]. It is important to consider the choice of needle gauge and length, as narrow gauge and longer needles may have a higher risk of breaking. Additionally, improper techniques such as redirecting the needle while the introducer is in place, removing the stylet, or using excessive force can increase the chances of complications. Analyzing the topic of successful neuraxial block, De Filho et al. identified several factors that can contribute to achieving success on the first attempt. These factors include the quality of the patient's anatomical landmarks, the adequacy of patient positioning, and the experience of the provider [12]. Since morbid obesity can present challenges during the placement of spinal anesthesia in obstetrics, Shah et al. proposed the use of ultrasound, which can help reduce the risk of complications in morbidly obese parturients [13]. Besides the use of ultrasound, Kaboré et al. recommended decreasing the number of puncture attempts to lower the risk of needle damage, as well as proper mobilization of the introducer, and even considering the choice of general anesthesia (GA) instead of spinal anesthesia in certain cases [9]. Lonnée et al. have proposed that in difficult cases we should use a needle-through-needle technique where the epidural needle guides the spinal needle to the dura [14].

It is important to be aware of the potential complications that can arise if a needle breaks, such as migration of the foreign body, infection, fibrosis of surrounding tissues, and neurological complications. The risk of neurological complications depends on the position of the needle and can lead to nerve damage, pain, infection, and CSF leakage. In case of needle breakage, the guidelines [5] recommend removing the needle as soon as possible by using medical imaging. If the patient does not have neurological symptoms, the removal can be done after the planned surgical procedure. However, if abnormal neurological symptoms are present, it is advised to seek urgent neurology consultation. Additionally, it is recommended to save the damaged needle, along with its batch number and specifications. In our case, a spinal needle introducer had been accidentally left in the patient's back and it got broken while the patient moved in the bed after the operation. We have found only one case report in the literature about a broken needle introducer, and it involved a metal needle shaft that got separated from a plastic hub because binding between these two was insufficient [8]. When this complication occurs, the patient should be informed and the case should be reported to the authorities. In one report, a patient's case was presented to a governmental organization for compensation claims as the patient thought they might have received an injury due to an error or omission in the treatment procedure. All such cases should be taken seriously, not only because of medical complications but also due to the possibility of lawsuits by patients for medical malpractice [14].

## Conclusions

Breaking of spinal needles and spinal needle introducers is a rare but serious complication. It is critical to identify the factors that may contribute to needle fracture during neuraxial anesthesia and implement the necessary precautions to prevent this complication. Prompt removal of the broken needle and reporting the incident to regulatory authorities are advised given the potential complications involved. Anesthesiologists should also be mindful of the potential for legal action by patients if this complication is not managed appropriately.
